# Body Image Change in Obese and Overweight Persons Enrolled in Weight Loss Intervention Programs: A Systematic Review and Meta-Analysis

**DOI:** 10.1371/journal.pone.0124036

**Published:** 2015-05-06

**Authors:** Hai-Lun Chao

**Affiliations:** Department of Health Care Administration, Chung-Hwa University of Medical Technology, Tainan, Taiwan; Cardiff University, UNITED KINGDOM

## Abstract

To report the results of a systematic review and meta-analysis examining whether weight loss interventions improve body image in obese/overweight individuals. Medline, Current Contents, and the Cochrane database were searched to identify studies involving obese/overweight adults who were enrolled in weight loss interventions in which body image was quantitatively assessed. Outcomes assessed were changes in body shape concern, body size dissatisfaction, and body satisfaction (intervention vs comparator/control group). Seven studies were included in the systematic review (4 in the meta-analysis). All but 1 study involved female participants only. The type of weight loss intervention varied between studies as did the type of control/comparator group. In 3 studies, there was no significant difference in body image outcomes, whereas in 4 studies, improvement in body image was significantly more pronounced in the intervention vs the control/comparator group. Meta-analysis revealed that improvements in body shape concern (standardized difference: -0.52; 95% confidence interval [CI]: -1.04 to 0.00), body size dissatisfaction (standardized difference: -0.66; 95% CI: -0.88 to -0.45), and body satisfaction (standardized difference: 0.74; 95% CI: 0.09 to 1.38) significantly favored the intervention over the comparator/control group (*P<*0.05). The results of this systematic review/meta-analysis lend support to the notion that weight loss interventions may improve body image. This is a noteworthy finding that has clear clinical applicability because body image affects psychological well-being and the ability of an individual to maintain weight loss. Future research should determine which weigh loss interventions are associated with optimal improvements in body image and maintenance of weight loss.

## Introduction

Obesity and overweight are significant problems in developed countries and an increasing problem in most undeveloped countries [1]. Of note, recent estimates indicate that approximately 35% of adults in the United States are obese [2]. High rates of obesity are concerning given the well-established link between obesity and various medical conditions, such as diabetes, high blood pressure/hypertension, dyslipidemia, cardiovascular disease, and sleep apnea [3]. Unsurprisingly, the medical care costs associated with obesity are immense [4]. As a consequence, research into the treatment of obesity and overweight is of considerable importance.

Obesity treatment typically comprises lifestyle changes, such as dietary modification and exercise interventions, that aim to reduce body weight. Such interventions may be effective in the short term; however, long-term maintenance of weight loss is often challenging for obese individuals. An additional factor that appears to warrant consideration when designing and assessing the effectiveness of weight loss interventions is body image, defined as “the multifaceted psychological experience of embodiment” [5]. Body image consists of two dimensions, evaluative and investment, which in turn comprise aspects of subjective (dis)satisfaction, cognitive distortions, affective reactions, behavioral avoidance, and perceptual inaccuracy [6,7]. These components can be assessed by various instruments designed to measure weight satisfaction, appearance satisfaction, body image investment, and size perception [6]. Poor body image and the consequent impact on psychological well-being is inextricably linked to obesity in many individuals [8]. Therefore, determining whether weight loss interventions affect body image in obese individuals is a worthwhile endeavor.

During the last five to six years, a number of individual studies have reported the effects of weight loss interventions on body image in overweight and obese people. To gain a more comprehensive understanding of this issue, we decided to perform a systematic review and meta-analysis of the literature to determine whether obese individuals experience improvements in body image while participating in targeted weight loss interventions.

## Methods

### Search Strategy

Medline, Current Contents, and the Cochrane database were searched using combinations of the following search terms: body image, obesity/obese, overweight, weight loss, weight management, physical activity, dietary behaviors, eating/dietary/nutrition, intervention/program/education. The search was carried out on 30 May 2013. The following outlines the strategy used in Medline. Filters activated were comparative study and clinical trial. 143 records were found using the following combination of search terms: (Obesity or obese or overweight) and (Body image) and (Weight loss or management) AND (body image) AND (weight loss). 69 records were found using the following combination of search terms: (((Obesity or obese or overweight) and (Body image) and (Weight loss or management)) and ((physical activity or exercise or dietary or eating or nutrition)) and ((intervention or program or education))). 64 duplicate records were found in both combinations. In total, 148 records (143+69–64) were found. This systematic review and meta-analysis was carried out in accordance with the PRISMA guidelines [9].

### Selection of Studies

Prospective or retrospective studies were considered for inclusion if they involved obese or overweight adults who were enrolled in weight loss interventions in which body image was quantitatively assessed and they included a comparator control group of obese or overweight adults. Studies were excluded if the participants had any co-existing chronic disease or if body image was not assessed quantitatively.

### Data Extraction and Quality assessment

Two independent reviewers extracted the data from eligible studies. A third reviewer was consulted for resolution of disagreement. The following information/data was extracted from each eligible study: first author and year of publication, type of intervention and comparator/control group, body mass index (BMI) of participants, duration of follow-up, tool(s) used for assessing body image, body image domains assessed, and body image scores at baseline and follow-up.

The extent of agreement between reviewers was determined by calculating coder drift as described by Cooper and colleagues [10]. Per case agreement was determined by dividing the number of variables coded the same by the total number of variables. Acceptance required a mean agreement of 0.90. The coder drift in this study was calculated to be 0.94, indicating an acceptable level of agreement.

The methodological quality of each study was assessed using the risk-of-bias assessment tool outlined in the Cochrane Handbook for Systematic Reviews of Interventions [11]. Two reviewers subjectively reviewed all studies and assigned a value of “low risk,” “high risk,” or “unclear” to the following: a) random sequence generation, b) allocation concealment, c) blinding (patients, personnel, and assessor), d) adequate assessment of each outcome, e) selective outcome reporting avoided, and f) if the analysis include an intention to treat analysis.

The outcomes of interest were body shape concern, body size dissatisfaction, and body satisfaction.

### Data Analysis

The standardized differences in means with 95% confidence intervals (CI) were calculated for each outcome for the intervention group and comparator group. Heterogeneity among the eligible studies was assessed by determining the Cochran Q and the I^2^ statistic. For the Q statistic, *P* < 0.10 was considered to indicate statistically significant heterogeneity. For the I^2^ statistic, which indicates the percentage of observed between-study variability due to heterogeneity rather than chance, no heterogeneity was indicated by 0 to 25%, moderate heterogeneity was indicated by 25 to 50%, large heterogeneity was indicated by 50 to 75%, and extreme heterogeneity was indicated by 75 to 100%. If either the Q statistic (*P* < 0.1) or I^2^ statistic (> 50%) demonstrated the existence of heterogeneity between studies, a random-effects model (DerSimonian—Laird method) of analysis was used. Otherwise, a fixed-effect model (Mantel-Haenszel method) of analysis was used. Pooled standardized differences in means for all three outcomes were calculated. A two-sided *P* value < 0.05 was taken to indicate statistical significance. All statistical analyses were performed using the statistical software Comprehensive Meta-Analysis, version 2.0 (Biostat, Englewood, NJ).

## Results

### Literature Search

**Fig 1:**
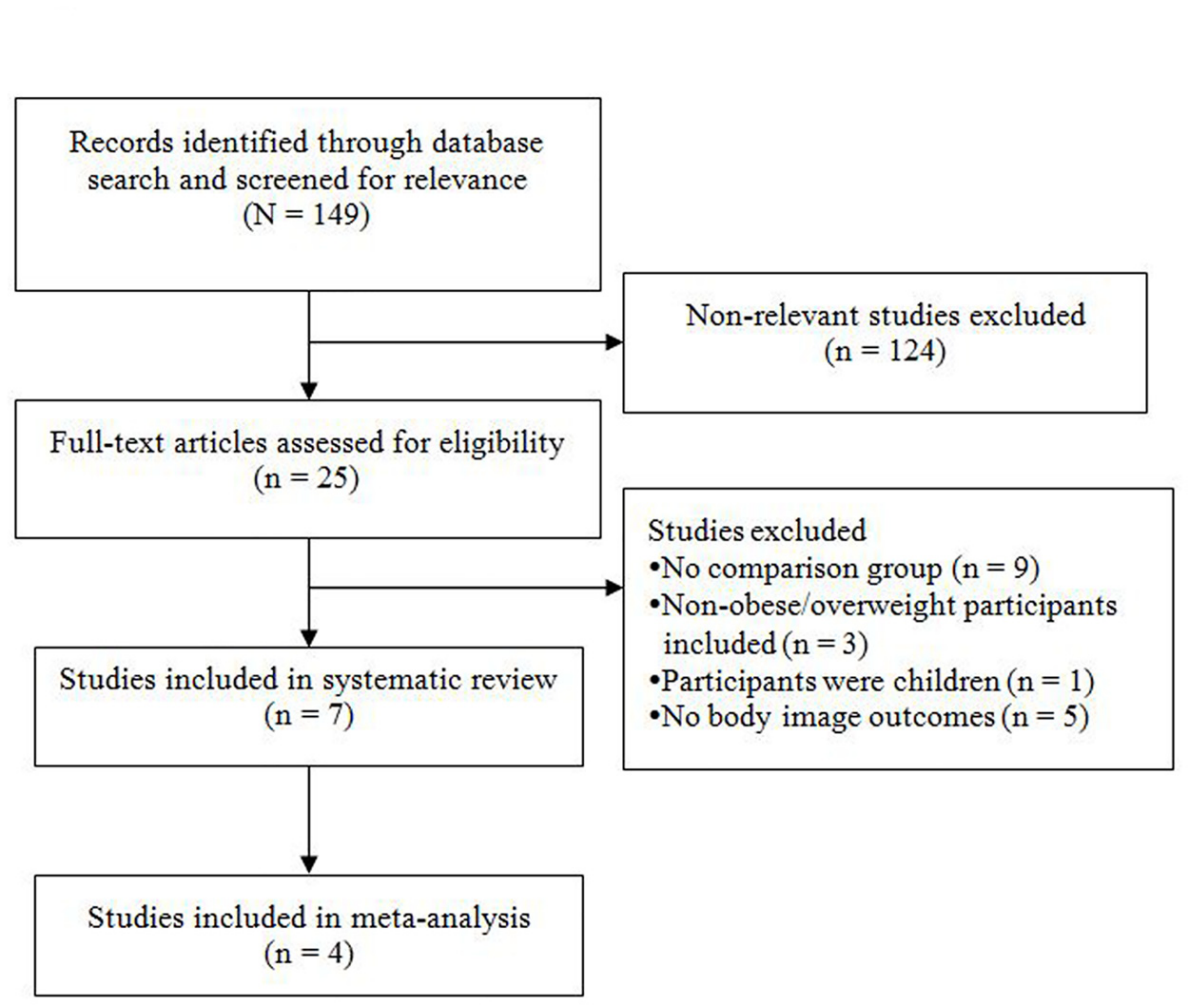
Flow diagram of study selection.

After the removal of 9 duplicate publications, a total of 149 unique articles were identified in the initial search. Of these, 124 did not report relevant body image outcomes and were excluded before full-text review. Of the remaining 25 articles that underwent full-text review, 7 were included in the systematic review and 4 were included in the meta-analysis. The most common reason for exclusion after full-text review was the lack of a treatment comparator group (Fig 1). The 3 studies not included in the meta-analyses either used unique tools to assess body image or did not report data in the form of mean and standard deviation.

### Study Characteristics

The characteristics of the 7 studies that met the eligibility criteria are summarized in Table 1. All studies except that reported by Munsch et al [12] involved females only. The study reported by Munsch et al [12] involved 92 females and 30 males. The type of weight loss interventions varied between studies. In the study reported by Crerand et al [13], participants attended meetings for 40 weeks during which they were recommended to follow specific dietary practice or received more general information. In the studies reported by Teixeira et al [14] and Palmeira et al [15], participants attended obesity treatment programs for 12 months or received general health education. The two studies reported by Annesi et al comprised 6 months of exercise in addition to advice on nutrition in comparison with no intervention [16] or 6 months of exercise in addition to cognitive behavioral support in comparison with exercise intervention alone [17]. In the study reported by Rapoport et al [18], participants received 10 weeks of CBT, which focused on the psychosocial costs and the physiological health risks of obesity, or standard CBT. In the study reported by Munsch et al [12], participants underwent a treatment program (duration not specified) for obesity, with an emphasis on lifestyle and eating behavior changes (at a general practice or a clinical center) or received non-specific information about weight loss. All participants in these studies were either overweight or obese, with BMI ranging from 25 to 45. The number of participants ranged from 31 to 144 (N = 597) in the main intervention group and from 17 to 88 (N = 334) in the comparator group. Different tools were used to measure body image between studies. The Body Shape Questionnaire was used to assess body shape concern in three studies [13–15], while the Body Image Assessment tool was used to assess body size dissatisfaction in two studies [14,15]. The Body Areas Satisfaction Scale was used to assess body satisfaction in two studies [16,17]. In the studies reported by Rapoport et al [18] and Munsch et al [12] unique measurement tools (in the context of this review) were used to assess body satisfaction and body image avoidance [18], and attractiveness/self confidence, accentuation of appearance, insecurity/concern, and physical/sexual misperception [12], respectively.

**Table 1:** Summary of basic characteristics and outcomes of studies included in the systematic review.

Author(Year)	Study Design	Comparison	BMI	Age (mean ± SD)	Follow-up	Measurement Tool	Body Image Domain	Body Image Score	
								Baseline	Follow-up
Crerand (2007) [12]	RCT	Meal replacement diet/balanced deficit diet (n = 84) vs. Non-dieting program (n = 39)	30–43	44.3±9.9	20 wk	BSQ	Body shape concern	100.5±26.2	-18.40%
				vs				vs	vs
				43.9±10.2				97.0±26.5	-15.90%
Teixeira (2010) [13]	RCT	Behavior change intervention (n = 106) vs. General health education (n = 88)	25–40	37.6±7.0 (all participants)	12 m	BSQ	Body shape concern	100.7±25.8	69.6±19.5
								vs	vs
								97.5±25.0	85.3±25.6
						BIA	Body size dissatisfaction	2.52±0.81	1.48±0.72
								vs	vs
								2.55±0.78	2.01±0.78
Palmeira(2009) [14]	NRCT	Behavior change intervention (n = 144) vs. General health education (n = 49)	25–40	39.0±6.6	12 m	BSQ	Body shape concern	96.5±27.7	76.1±26.4
				vs				vs	vs
				36.6±6.8				99.0±22.1	85.2±24.2
						BIA	Body size dissatisfaction	2.4±0.7	1.6±0.7
								vs	vs
								2.5±0.8	2.2±0.8
Annesi(2008) [15]	RCT	Exercise + nutrition support (n = 59) vs.	≥ 30	43.9±9.6 (all participants)	6 m	BASS	Body satisfaction	17.95±3.73	22.23±4.59
		No intervention (n = 43)						vs	vs
								16.51±3.94	16.27±4.32
Annesi (2010) [16]	RCT	Exercise + cognitive behavioral support (n = 68) vs.	30–45	42.2±10.5 (all participants)	24 wk	BASS	Body satisfaction	1.99±0.47	2.49±0.60
		Exercise + standard instruction (n = 66)						vs	vs
								2.14±0.50	2.40±0.66
Rapoport (2000) [17]	RCT	Modified CBT (n = 31)	≥ 28	NA	52 wk	BSS	Body satisfaction	47±11	40±10
		vs						vs	vs
		Standard CBT (n = 32)						44±12	39±12
						BIAQ	Body image avoidance	36±11	32±10
								vs	vs
								33±11	29±8
Munsch (2003) [11]	RCT	CBT (GP) (n = 53) vs.	≥ 30	female: 49 ± 12; male: 45 ± 14 vs	1 y	Body image questionnaire	Attractiveness/ self-confidence	7.0±3.7	8.6±3.8
		Control (non-specific advice) (n = 17) vs.		female: 49 ± 10; male: 49 ± 10 vs				vs	vs
		CBT (clinical center) (n = 52)		female: 46 ± 13; male: 37 ± 13				6.0±5.2	5.2±6.5
								vs	vs
								5.2±2.6	8.1±4.1
							Accentuation of appearance	6.7±2.2	6.6±2.5
								vs	vs
								5.8±2.5	6.0±1.7
								vs	vs
								6.2±3.1	6.7±2.1
							Insecurity/concern	4.8±1.9	3.7±2.0
								vs	vs
								5.8±4.0	5.7±3.9
								vs	vs
								4.8±3.2	4.8±3.3
							Physical/sexual misperception	2.0±1.4	1.7±1.5
								vs	vs
								3.7±1.8	3.8±2.5
								vs	vs
								1.4±1.5	1.3±1.9

Abbreviations: RCT, randomized clinical trial; NRCT, non-randomized clinical trial; BSQ, Body Shape Questionnaire; BCS, Body Cathexis Scale; BIA, Body Image Assessment; BASS, Body Areas Satisfaction Scale; BSS, Body Satisfaction Scale; BIAQ, Body-Image Avoidance; m, month; y, year; wk, week. Questionnaire; CBT, Cognitive Behavioral Therapy; GP, general practitioner; NA, no data available.

### Study Outcomes and Quality Assessment

The individual study outcomes are summarized in Table 1. Crerand et al [13] reported that body shape concern decreased (indicating improvement) in both groups after the intervention; however, there was no significant between group difference in the extent of the decrease. Teixeira et al [14] reported that although body shape concern and body size dissatisfaction decreased in both groups after the intervention, the extent of the decrease was significantly more pronounced in the behavior change intervention group compared with the general health (comparator) education group. Palmeira et al [15] reported similar findings for body size dissatisfaction, but found no between group difference in body shape concern after the intervention. In two studies, Annesi et al reported that body satisfaction significantly increased (indicating improvement) in the main intervention group but not in the comparator group [16] or that the extent of the increase was more pronounced in the main intervention group [17]. Rapoport et al [18] reported that body satisfaction and body image avoidance significantly increased (indicating improvement) in both groups; however, there was no significant difference between groups. Munsch et al [12] reported that attractiveness/self confidence significantly increased (indicating improvement) in the CBT groups, but not in the control group. No other measures of body image changed significantly in any of the groups.

**Fig 2:**
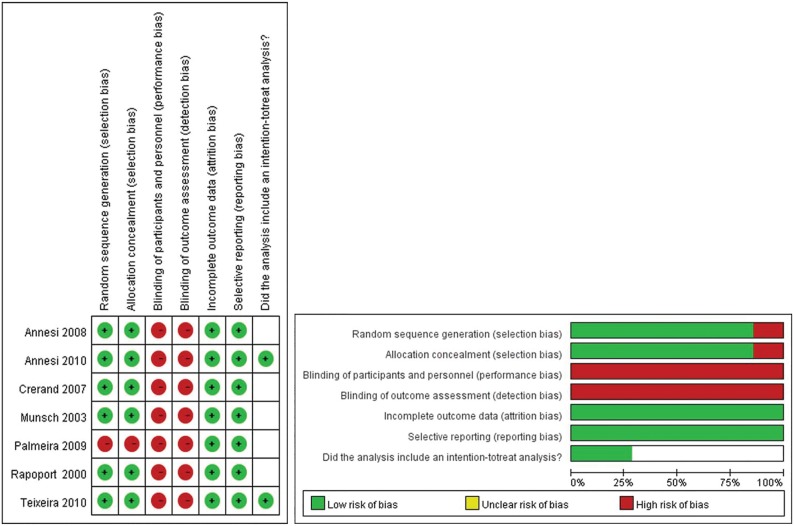
Quality assessment of included studies: Risk of bias (A) and overall assessment of risk of bias (B).

The “risk of bias” summary is presented in Fig 2A, and an overall assessment of risk of bias is presented in Fig 2B. All studies except that reported by Palmeira et al [15] had appropriate random sequence and allocation concealment. Neither participants nor outcome assessors were blinded to treatment in any of the studies. All studies had a low risk of attrition and reporting bias. Intention-to-treat analysis was only used in 2 studies [10, 13].

### Meta-analysis

Two studies were included in the meta-analyses of body shape concern [14,15], body size dissatisfaction [14,15], and body satisfaction [16,17].

#### Body Shape Concern

**Fig 3:**
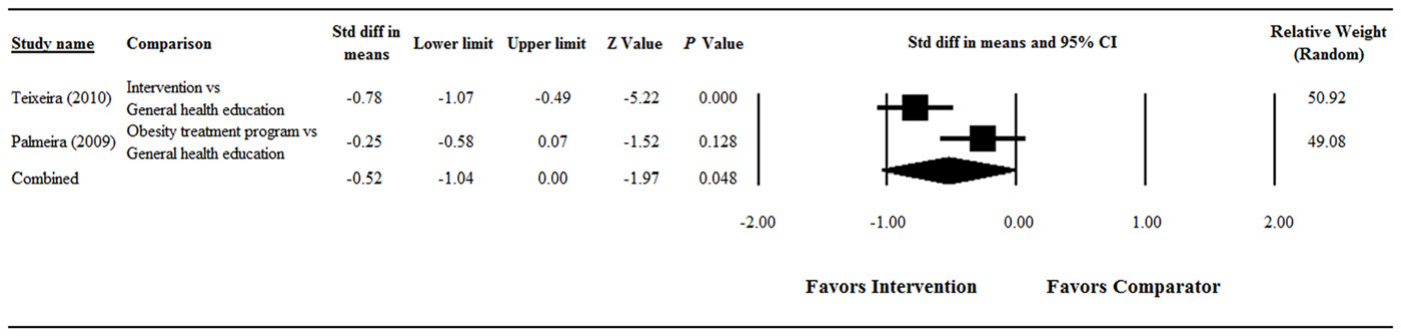
Forest plot showing results of the meta-analysis of body shape concern scores among obese/overweight participants. Abbreviations: Std diff, standardized difference; CI, confidence interval.

Significant heterogeneity was found when data from the two studies were pooled (Q = 5.58, df = 1, *P* = 0.018; I^2^ = 82.09%); therefore, a random-effects model of analysis was used (Fig 3). The overall analysis showed the change in body shape concern significantly favored the main intervention group over the comparator group (pooled standardized difference in means = -0.52, 95% CI = -1.04 to 0.00, Z = -1.97; *P* = 0.048).

#### Body Size Dissatisfaction

**Fig 4:**
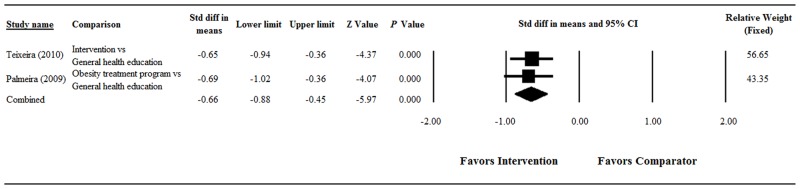
Forest plot showing results of the meta-analysis of body size dissatisfaction scores among obese/overweight participants. Abbreviations: Std diff, standardized difference; CI, confidence interval.

There was no evidence of significant heterogeneity when data from the two studies were pooled (Q = 0.04, df = 1, *P* = 0.851; I^2^ = 0%); therefore, a fixed-effect model of analysis was used (Fig 4). The overall analysis showed that the change in body size dissatisfaction significantly favored the main intervention group over the comparator group (pooled standardized difference in means = -0.66, 95% CI = -0.88 to -0.45, Z = -5.97; *P* < 0.001).

#### Body Satisfaction

**Fig 5:**
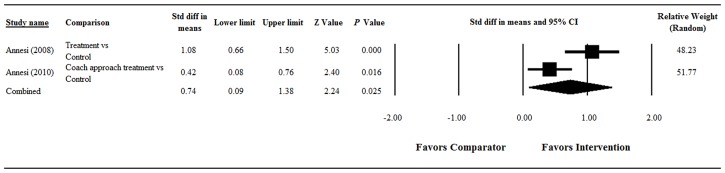
Forest plot showing results of the meta-analysis of body satisfaction scores among obese/overweight participants. Abbreviations: Std diff, standardized difference; CI, confidence interval.

Note: As each meta-analysis included only two studies, we did not perform sensitivity analysis or assess publication bias.

Significant heterogeneity was found when data from the two studies were pooled (Q = 5.68, df = 1, *P* = 0.017; I^2^ = 82.38%); therefore, a random-effects model of analysis was used (Fig 5). The overall analysis showed that the change in body satisfaction significantly favored the main intervention group over the comparator group (pooled standardized difference in means = 0.74, 95% CI = 0.09 to 1.38, Z = 2.24; *P* = 0.025).

## Discussion

In this systematic review and meta-analysis, we examined changes in body image among obese/overweight individuals who were enrolled in weight loss intervention programs. A total of 7 studies, involving 931 participants, met the eligibility criteria for inclusion. Four of these studies included appropriate data for meta-analysis. Of interest, 4 of the studies included in the systematic review reported that the intervention significantly improved body image to a greater extent than the control/comparator intervention. Meta-analysis revealed that improvements in body shape concerns, body size dissatisfaction, and body satisfaction significantly favored the intervention over the comparator group.

The findings of our meta-analyses and systematic review suggest that weigh loss intervention programs may help improve body image among obese/overweight persons. This is of interest given that body image can affect psychological well-being [8] and indeed weight loss/maintenance of weight loss [19,20]. Various other studies (not included in our systematic review/meta-analysis because they did not include a comparator group) involving obese/overweight adult women have also found improvements in body image on the completion of weight loss intervention programs [21–23]. Of note, we found that multiple measures of body image, namely body shape concerns, body size dissatisfaction, and body satisfaction improved to a greater extent with the active intervention. Taken together, these measures provide an indication of both dimensions of body image, evaluative and investment; therefore, weight loss intervention programs may be effective for improving body image as a whole rather one dimension more than the other. Nevertheless, it would be interesting to determine in future research which dimensions of body image are more or less affected by certain interventions and how these changes (or lack thereof) affect weight loss and maintenance of weight loss. To this end, research examining the relationship between body image and self-eating regulation for weight loss suggests that improvements in the investment dimension of body image may be more important for improving self-eating regulation than the evaluative dimension [21]. Likewise, the investment dimension of body image appears to be more significantly affected by physical activity than the evaluative dimension [24]. Whether or not targeted weight loss interventions other than self-eating regulation have similarly differential effects on the dimensions of body image remains to be determined in appropriately designed studies.

Not all of the studies included in our review reported that the main weight loss intervention was superior to the comparator intervention for improving body image. The lack of homogeneity between studies may account for this disparity. In particular, there was a large variability between the studies, namely regarding the type of intervention and comparator groups, the length of follow-up, the type of body image assessment tools used, and the component(s) of body image assessed. The lack of homogeneity between studies in the type of intervention used may be critical because the variation in effectiveness may be dependent on intervention type. In fact, no two studies used the same weight loss intervention program. The type of intervention ranged from diet, to exercise, to CBT. It must be noted, however, that in the studies in which significant between group differences were not apparent [13,15,18], improvements from baseline were detected for both the intervention group and the comparator group. Hence, the findings from these studies suggest that some form of intervention (e.g. general health education or non-dieting program) may also have beneficial effects on body image in obese/overweight adults. Of note, only one of the studies [16] included in our review involved a true control (ie, no intervention) comparator group. Clearly, there is a need for well-designed randomized controlled trials to identify interventions that optimize improvements in body image.

Our systematic review and meta-analysis has a number of limitations. Firstly, only a relatively small number of studies were included, particularly in the meta-analysis. Clearly, the small number of studies raises the possibility of publication bias and limits the power of analysis and, hence, the capacity to detect definitive effects of the intervention. Secondly, the studies included in the analyses for each body image outcome were from the same group of researchers. Hence, potential investigator bias cannot be discounted. Further, the wider generalizability of the findings from this group of researchers requires confirmation. Thirdly, as already noted, there was a distinct lack of homogeneity between the studies included, with regards to various factors, most notably both the active and comparator/control interventions and the tools used to assess body image. Further, both within and between some studies, there was considerable variability in the BMI range of participants. This lack of homogeneity and variability may have affected the pattern of change in body image and hence our findings. Fourthly, neither the participants nor the outcome assessors were blinded in any of the studies. This lack of blinding introduces the possibility of bias (from both a participant and assessor perspective), but is really something that is very difficult, if not impossible, to implement in studies of this nature. Finally, all but one study [12] involved females only; hence, the applicability of the results to obese males is uncertain. In light of these limitations, the results of our meta-analysis, in particular, must be interpreted with some degree of caution. Nevertheless, all the studies included in our systematic review/meta-analysis were prospective and randomized in design, thereby strengthening the results reported herein.

## Conclusions

In conclusion, the findings of our systematic review and meta-analysis support the notion that weight loss interventions may improve body image in obese/overweight adults. This is important given that body image is an important mediator of psychological well-being and the capacity for an individual to maintain weight loss. We do acknowledge, however, that the current body of available evidence is far from conclusive. Clearly, further research, in large-scale randomized controlled trials, is needed to determine whether certain weight loss interventions facilitate optimal outcomes in terms of both improving body image and facilitating and maintaining weight loss.

## Supporting Information

S1 PRISMA Checklist S1 PRISMA 2009 Checklist. (DOC)

S1 PRISMA Flow Diagram S1 PRISMA 2009 Flow Diagram. (DOC)
