# Supplementary material for: Body Image Change in Obese and Overweight Persons Enrolled in Weight Loss Intervention Programs: A Systematic Review and Meta-Analysis
Source: PLoS One. 2015 May 6;10(5):e0124036. doi: 10.1371/journal.pone.0124036 (PMC4422747; doi:10.1371/journal.pone.0124036)
Supplement: S1 PRISMA Flow Diagram — (DOC) [file pone.0124036.s002.doc]

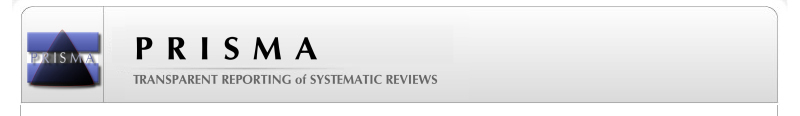
**PRISMA 2009 Flow Diagram**

**Screening**

**Included**

**Eligibility**

**Identification**

Records identified through database searching
(n = 149)

Additional records identified through other sources
(n = 0)

Records after duplicates removed
(n = 149)

Records screened
(n = 149)

Records excluded
(n = 123)

Full-text articles assessed for eligibility
(n = 26)

Full-text articles excluded, with reasons
(n = 18)

•No comparison group (n = 9)

•Non-obese/overweight participants included (n = 3)

•Participants were children (n = 1)

•Non-relevant outcomes (n = 5)

Studies included in qualitative synthesis
(n = 7)

Studies included in quantitative synthesis (meta-analysis)
(n = 4)
